# Prospective clinical study to evaluate an oscillometric blood pressure monitor in pet rabbits

**DOI:** 10.1186/s12917-018-1369-4

**Published:** 2018-02-27

**Authors:** Luca Bellini, Irene A. Veladiano, Magdalena Schrank, Matteo Candaten, Antonio Mollo

**Affiliations:** 1 Veterinary Teaching Hospital, University of Padua, Viale dell’Università 16, 35020 Legnaro, PD Italy; 2 Department of Animal Medicine, Production and Health, University of Padua, Viale dell’Università 16, 35020 Legnaro, PD Italy

**Keywords:** Rabbits, Arterial blood pressure, Oscillometric method, Isoflurane, Trending ability

## Abstract

**Background:**

Rabbits are particularly sensitive to develop hypotension during sedation or anaesthesia. Values of systolic or mean non-invasive arterial blood pressure below 80 or 60 mmHg respectively are common under anaesthesia despite an ongoing surgery. A reliable method of monitoring arterial blood pressure is extremely important, although invasive technique is not always possible due to the anatomy and dimension of the artery. The aim of this study was to evaluate the agreement between a new oscillometric device for non-invasive arterial blood pressure measurement and the invasive method. Moreover the trending ability of the device, ability to identify changes in the same direction with the invasive methods, was evaluated as well as the sensibility of the device in identifying hypotension arbitrarily defined as invasive arterial blood pressure below 80 or 60 mmHg.

**Results:**

Bland-Altman analysis for repeated measurements showed a poor agreement between the two methods; the oscillometric device overestimated the invasive arterial blood pressure, particularly at high arterial pressure values. The same analysis repeated considering oscillometric measurement that match invasive mean pressure lower or equal to 60 mmHg showed a decrease in biases and limits of agreement between methods. The trending ability of the device, evaluated with both the 4-quadrant plot and the polar plot was poor. Concordance rate of mean arterial blood pressure was higher than systolic and diastolic pressure although inferior to 90%. The sensibility of the device in detecting hypotension defined as systolic or mean invasive arterial blood pressure lower than 80 or 60 mmHg was superior for mean oscillometric pressure rather than systolic. A sensitivity of 92% was achieved with an oscillometric measurement for mean pressure below 65 mmHg instead of 60 mmHg. Non-invasive systolic blood pressure is less sensitive as indicator of hypotension regardless of the cutoff limit considered.

**Conclusions:**

Although mean invasive arterial blood pressure is overestimated by the device, the sensitivity of this non-invasive oscillometric monitor in detecting invasive mean pressure below 60 mmHg is acceptable but a cutoff value of 65 mmHg needs to be used.

## Background

Anaesthesia related mortality at 48 h post-procedure is higher in rabbits than in dogs and cats [1]. Rabbits often suffer from subclinical cardiopulmonary diseases that may be undetected before anaesthesia [1]. Moreover sedatives and anaesthetics may cause cardiovascular depression [2–4] that may lead to hypotension with organ hypoperfusion and ischemic injuries that may precipitate subclinical diseases. Harvey et al. observed that almost 92% of rabbits showed systolic or mean arterial blood pressure lower than 80 or 60 mmHg respectively at a surgical plane of anaesthesia [4].

Direct invasive arterial blood pressure (IBP) measurement is considered the “gold standard” for arterial blood pressure measurement. This technique requires an arterial catheterization that may be technically difficult in small animals and it may lead to blood extravasation with haematoma and risk of ear necrosis [5]. The central auricular artery is often used for IBP measurement although the size of some breeds of pet rabbits limits the placement of a catheter in that artery. Non-invasive arterial blood pressure (NIBP) measurement methods may overcome some of these limitations and the agreement between these methods has been evaluated.

The Doppler technique showed good agreement with systolic IBP measurements and it can be used to monitor arterial pressure [3, 4], but does not provide information of mean and diastolic blood pressure. Moreover to improve the signal quality, often the skin over the metatarsal artery is clipped and this can predispose to skin irritation and pododermatitis [6].

Traditional oscillometric techniques that use equipment designed for human use becomes inaccurate at fast pulse rate or with pulse oscillation of small magnitude during hypotension or with alpha-2 agonist sedatives [5, 7]. Moreover the same authors suggested a low accuracy of such a devices in animal weighing lower than 5–7 kg [5]. The size of the cuff is critical and may add a source of error during oscillometric and Doppler methods. The width of the cuff should be approximately 40% of limb circumference but in small rabbits may not be available [4, 5]. Additionally, specific algorithm are introduced by manufacturers of NIBP devices to estimate SAP and DAP and they may be not accurate in all species, haemodynamic conditions and cuff location [7–9].

Recently, a new oscillometric device has become available for arterial blood pressure monitoring in dogs and cats. The device is provided with a ruler to allow selection of the most appropriate cuff size for the particular animal.

The aim of this study is to compare the new NIBP oscillometric device with the IBP measured from the auricular artery in anesthetized rabbits undergoing elective neutering. A secondary aim was to study the trending ability of the device and its sensitivity and specificity in detecting systolic and mean IBP lower than 80 and 60 mmHg respectively.

## Methods

Internal institutional ethics committee approved the study and owner’s consent was obtained before each procedure. Nineteen ASA 1–2 cross-breed pet rabbits of both sexes (7 male, 12 female) weighing between 2.5 and 4.6 kg, older than 6 months undergoing elective neutering were enrolled in a prospective clinical study.

### Anaesthesia

The animals were considered healthy based on the medical history and pre-anaesthetic clinical exam. Rabbits had free access to water and food until a combination of dexmedetomidine 50 μg/kg (Dexdomitor; Orion Corporation, Espoo, Finland) and midazolam (Midazolam-hameln; Hospira Italia, Naples, Italy) 0.6 mg/kg was administered into the lumbar muscles (total volume 0.22 ml/kg) to obtain sedation. As the rabbits lost the righting reflex the external pinnae of the left ear and the dorsal aspect of the right foreleg were clipped. A 24-gauge over-the-needle intravenous (IV) catheter was aseptically inserted in the cephalic vein. After IV access was achieved, 0.05 mg/kg of buprenorphine (Buprenodale 0.3 mg/ml; Dechra Ltd., Skipton, UK) was administered together with a subcutaneous injection of 1 mg/kg of meloxicam (Metacam; Boehringer Ingelheim, Milan, Italy) and 5 mg/kg of enrofloxacin (Baytril 25 mg/ml injection solution; Bayer Spa, Milan, Italy). Anaesthesia was induced with IV propofol (Vetofol 10 mg/ml, Norbrook Laboratories Ltd., Monaghan, Northern Ireland) and airways were secured either with an endotracheal tube or a laryngeal mask in 9 and 10 rabbits respectively. The animals were placed in dorsal recumbency and anaesthesia was maintained with isoflurane (Isoflo, Abbott Laboratories Ltd., Maidenhead, UK) in a mixture of oxygen and air (FiO_2_ = 0.5) via a circle breathing system with paediatric tubes. The end-tidal concentration of isoflurane was adjusted to maintain an adequate surgical plane of anaesthesia while minimizing the detrimental cardiovascular effects of volatile agent. To maintain a normothermic range between 38° and 39 °C an electric heating pad (Pet-Mat; Dale Ecotech Pty Ltd., Milsons Point, Australia) was placed under the rabbits. Pulse rate, pulse oximetry, IBP, respiratory rate, end-tidal concentration of isoflurane and carbon dioxide were monitored with a portable pulseoximeter (EDAN VE-H100B, Edan USA; San Diego, CA, USA) and a multiparameter monitor (Datex S/5; GE Healthcare; Helsinki, Finland); a mixture of 50:50 lactate Ringer’s solution and 5% dextrose was infused at 5 ml/kg/h IV. After surgery all monitoring devices were removed and the rabbits received subcutaneously 0.5 mg/kg of atipamezole (Antisedan, Orion Corporation, Espoo, Finland). Heart rate, respiratory rate and signs of pain were evaluated in the recovery period until discharge.

### Invasive arterial blood pressure measurement

To measure the invasive arterial blood pressure a 24-gauge Teflon catheter (Delta Ven 1; DeltaMed Spa, Viadana, Italy) was inserted aseptically in the median portion of central auricular artery and it was secured with tape. If the artery was not catheterized at the first attempt the animal was removed from the study and arterial blood pressure was recorded by a Doppler ultrasound method as previously reported (Bellini et al. [3]). The arterial catheter was connected to a pressure transducer by a 150 cm long non-compliant tubing (TruWaveTM pressure monitoring set; Edwards Lifesciences, Irvine, CA, USA) filled with heparinized saline (5 IU/ml) and connected to the multiparameter monitor and a pressurized (300 mmHg) bag containing saline solution. The IBP was calibrated against a bourdon manometer (Tycos TR-2 Hand Aneroids, Welsh Allyn Ltd., Aston Abbotts, UK) at the beginning of each day. The transducer was zeroed at the level of the manubrium of the sternum once the rabbit was placed in dorsal recumbency. Dynamic response test was performed at the beginning of each procedure. Damping coefficient and natural frequency were measured according to the formula reported in literature [10].

### Oscillometric arterial blood pressure measurement

A new oscillometric device (PetTrust Blood Pressure Monitor, BioCare, Aster Electrical Co. Ltd., Taiwan) was evaluated. The cuff was placed on the anterior forelimb proximal to the carpus once the animal was in dorsal recumbency; the correct size of the cuff was obtained by the ruler provided by the manufacturer and the inflation cuff line was orientated toward the distal part of the forelimb. The legs were loosely tied with a bandage role. Every 5 min 2 consecutive readings were taken and if the variability was less than 20% the data were averaged otherwise a third reading was done and the values that had the small variance were used [11].

### Statistical analysis

Mean bias, precision defined as standard deviation (SD) of the bias and limits of agreement (mean bias ± 1.96 × SD) for systolic, diastolic and mean arterial blood pressure measured with IBP and NIBP was assessed by the method proposed by Bland and Altman for multiple observations per individual [12]. Analysis was performed on all paired measurements and repeated including only pair measurements matching non-invasive SAP and MAP lower than 90 and 65 mmHg respectively. An acceptable agreement between the two methods was considered achieved with a mean bias of less than 10 mmHg and a precision of less than 15 mmHg, that represent one of the criteria proposed by ACVIM Hypertension Consensus and Veterinary Blood Pressure Society, to validate a blood pressure device in dogs and cats [11]. The goodness of agreement between methods was also evaluated based on the standard of the Association for the Advancement of Medical Instrumentation (AAMI), which requires a mean bias less than 5 mmHg and a precision of less than 10 mmHg.

The “trending ability” of the oscillometric method in tracking dependable changes with the invasive method was evaluated with a four-quadrant plot and a polar plot as reported previously [13]. Both analysis were repeated for SAP, DAP and MAP; the concordance rate was calculated as the percentage of data points outside the exclusion zone representing 5% or 10% changes in arterial blood pressure, in the upper right and lower left quadrant over the data points in all quadrants. Data points, which lay in an exclusion zone of 5 and 10%, are suggesting small variations in arterial blood pressure, depending rather on the precision of the device than being a reflection of real changes in haemodynamics [14]. A concordance rate over 90% was judged indicative of good trending ability [13, 15]. The polar plot was used to quantify the trending ability that was considered adequate if mean polar angle and radial limit of agreement were lower than 5° and ± 30° respectively [13–15].

Intraoperative hypotension was arbitrary defined as invasive SAP or MAP lower than 80 and/or 60 mmHg respectively [4]. Sensitivity, specificity, positive predictive value and negative predictive value of the device in detecting hypotension were calculated considering different cutoff values. Criteria to define hypotension were non-invasive SAP of 80 or 90 mmHg and/or non-invasive MAP of 60 or 65 mmHg. Sensitivity was calculated as the number of true hypotension measurements detected with the oscillometric methods divided by the number of all hypotensive measurements obtained with the invasive method. Specificity was calculated as the number of true non-hypotension measurements detected with the oscillometric method divided by the number of all non-hypotensive measurement obtained with the invasive method. The positive predictive value was calculated as the number of true hypotensive measurements obtained with the non-invasive method divided by the number of all hypotensive measurements obtained with the non-invasive method. The negative predictive value was calculated as the number of true non-hypotension measurements detected with the oscillometric method divided by the number of all non-hypotensive measurements obtained with the invasive method.

The number of animals used was estimated on the base of the criteria proposed by ACVIM Hypertension Consensus and Veterinary Blood Pressure Society [11]. To obtain a minimum of 150 readings, at least 15 rabbits would be necessary with at least 10 matched blood pressure measurements; to cover potential missed readings due to equipment failure or dropout animals due to unsuccessful artery catheterization, 19 rabbits were enrolled.

Graphpad (GraphPad Software, La Jolla, CA, USA), Matlab R2017a (The MathWorks Inc., Natick, MA, USA), MedCalc v12 (MedCalc Software bvba, Ostend, Belgium) and Microsoft Office Excel 2011 (Microsoft Corporation, Redmond, WA, USA) were used for the statistical analysis and to plot the data. Data normally distributed are expressed as mean ± SD otherwise as median (min-max).

## Results

One rabbit was excluded because arterial catheter insertion was unsuccessful; in the remaining 18 rabbits, 172-paired measurements were collected and analysed. None of the rabbits presented postoperative complication due to arterial catheterization. The dose of propofol was 1.2 (0.0–6.0) mg and anaesthesia time was between 30 and 60 min. Natural frequency and damping coefficient were 29 ± 7 Hz and 0.28 ± 0.08 respectively and an adequate dynamic response was obtained for all rabbits. The normothermic range was maintained in all animals, with a mean rectal temperature at recovery of 38.5 ± 0.9 °C.

**Fig. 1:**
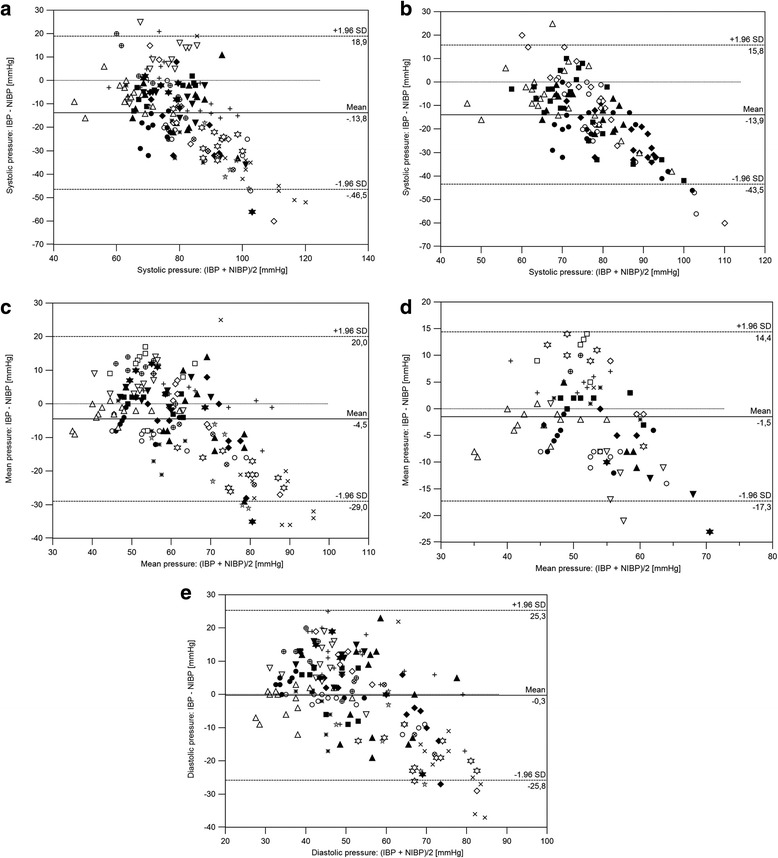
Bland-Altman plot for multiple comparisons for systolic (**a, b**), mean (**c, d**) and diastolic (**e**) arterial blood pressure measured in 18 pet rabbits undergoing elective neutering surgery. Bland-Altman analysis was performed on all the pair data (**a, c, e**) or only on pair measurements that had a systolic or mean invasive arterial blood pressure lower than 80 and 60 mmHg respectively (**b, d**). Solid line indicates mean bias, the two broken lines represent the limits of agreement; the dotted line is the line of equality

Results of Bland-Altman analysis of matched values for IBP over NIBP are shown in Table 1 and Fig. 1. Considering the overall values IBP was overestimated by the NIBP with a negative slope and a wide limits of agreement of approximately 50 mmHg for MAP and DAP. Repeating the analysis with pairs that included invasive SAP and MAP lower or equal to 80 and 60 mmHg respectively, resulted in a reduction of bias, standard deviation and limit of agreements compare to the previous analysis only for the mean arterial pressure (Fig. 1b and d). Both MAP and DAP met the requirements to define good agreement based on the limit of the ACVIM standard. Considering the AAMI standard instead, only pair measurements matching non-invasive MAP lower than 65 mmHg, showed good agreement. Agreement between methods was not adequate for SAP.

**Table 1:** Statistical summary and results of Bland-Altman analysis for multiple observations in 19 anaesthetized rabbits

	Systolic	Mean	Diastolic
IBP (mmHg)	74 ± 10	59 ± 10	52 ± 11
NIBP (mmHg)	87 ± 19	64 ± 18	52 ± 18
Bland-Altman agreement analysis			
Mean bias (mmHg)			
Overall	−13.8	−4.5	−0.3
Hypotension	−13.9	−1.5	–
Precision (mmHg)			
Overall	16.7	12.5	13.0
Hypotension	15.1	8.1	–
Limits of agreement (mmHg)			
Overall	−46.5 to 18.9	−29.0 to 20.0	− 25.8 to 25.3
Hypotension	− 43.5 to 15.8	−14.4 to 17.3	–

Systolic, mean and diastolic arterial pressure measured with an oscillometric method were compared to an arterial blood pressure measured invasively by central ear artery cannulation. Overall pair include all the value measured while the hypotension subgroup include all the pair measurements that had a systolic or mean invasive arterial blood pressure lower than 80 and 60 mmHg respectively. *IBP* invasive arterial blood pressure; *NIBP* non-invasive arterial blood pressure. Values are expressed as mean ± standard deviation

**Fig. 2:**
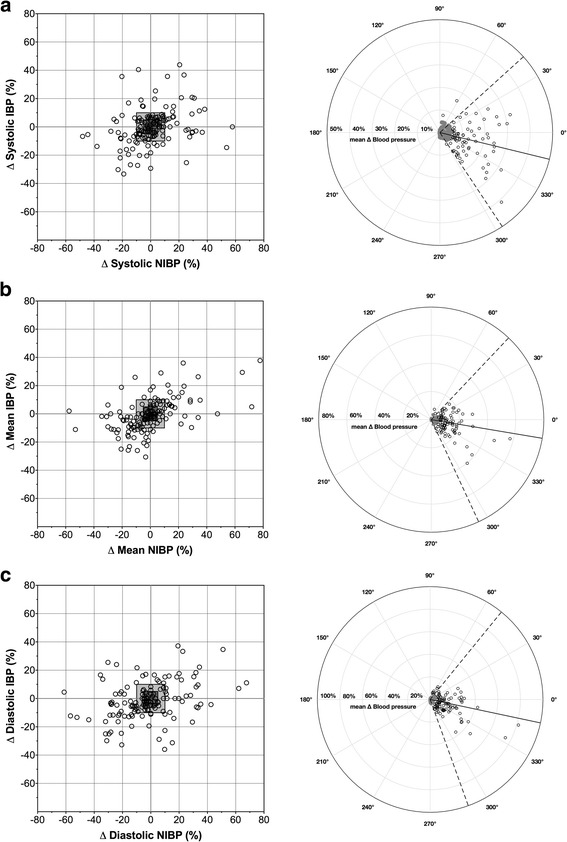
Four-quadrant (left) and polar plot (right) for systolic (**a**), mean (**b**) and diastolic (**c**) arterial blood pressure measured in 18 pet rabbits undergoing elective neutering surgery. In the concordance plot, each point represents a percentage changes in arterial blood pressure measured with an oscillometric device (∆ NIBP) or invasively (∆ IBP). In the 4-quadrant plot the grey areas limit the 10% and 5% exclusion zone where percentage changes in arterial blood pressure are ≤10% and 5% respectively. Dots in the upper left or lower right quadrant represent inaccurate trending between methods. The positive half circle polar plots show the values for the mean changes of arterial blood pressure measurement obtained non-invasively and invasively. The distance of each point from the center represents the mean change in arterial blood pressure calculated as absolute value of ∆ NIBP+∆ IBP/2; the angle with the horizontal axis shows the concordance of the methods. Solid line defines the mean angular bias and the dotted lines the upper and lower radial limit of agreement (bias ±1.96 × standard deviation) obtained for the data points in black. The grey points represent arterial blood pressure changes ≤5% (exclusion zone 5%) and were excluded from the analysis. Good agreement between the two methods is considered with a mean angular bias and a radial limit of agreement < 5° and < 30° respectively

The analysis of the trending ability is reported in Table 2. The changes in the oscillometric device poorly reflect the direction of changes of IBP. Independent of the exclusion zone the concordance rate for MAP, although lower than 90%, was higher than that of SAP and DAP (Table 2). The polar plot analysis showed a suboptimal trending ability with mean polar angles higher than 5° and a radial limits of agreement wider than ± 30° (Table 2 and Fig. 2).

**Table 2:** Trending ability with concordance and polar plot data for 18 anaesthetized rabbits

	Systolic	Mean	Diastolic
Concordance rate			
Exclusion zone 5%	67%	84%	67%
Exclusion zone 10%	65%	87%	72%
Polar plot analysis (n)	82	100	90
Mean polar angle			
Exclusion zone 5%	−13.7°	−9.5°	− 12.0°
Standard deviation	28.0°	28.1°	31.4°
Radial limits of agreement			
Exclusion zone 5%	− 56.4° to 42.2°	−65.1° to 46.6°	−70.2° to 50.8°

Data points representing percentage changes lower than 5% (exclusion zone 5%) or 10% (exclusion zone 10%) were not included in the analysis. n: number of observations

Sensitivity of the device to identify hypotension was higher if the cutoff for the corresponding non-invasive pressure limits were set at 90 and 65 mmHg respectively. Considering those cutoff values the specificity decreased. Positive predictive value for systolic pressure was higher than that of mean blood pressure while the opposite was observed for the negative predictive value (Table 3).

**Table 3:** Ability of the oscillometric monitor in detecting systolic or mean invasive pressure lower than 80 or 60 mmHg

	Sensitivity	Specificity	Positive predictive value	Negative predictive value
Systolic arterial blood pressure				
Cutoff of 80 mmHg for NIBP	46.3% (37.3%–5.56%)	77.6% (63.4%–88.2%)	83.8% (72.9%–91.6%)	36.5% (27.3%–46.6%)
Cutoff of 90 mmHg for NIBP	74.0% (65.3%–81.5%)	57.1% (42.2%–71.2%)	81.3% (72.8%–88.0%)	46.7% (33.7%–60.0%)
Mean arterial blood pressure				
Cutoff 60 of mmHg for NIBP	73.8% (63.0%–82.8%)	78.4% (68.4%–86.5%)	76.5% (65.8%–85.3%)	75.8% (65.7%–84.2%)
Cutoff 65 of mmHg for NIBP	91.8% (83.6%–96.6%)	61.4% (50.4%–71.6%)	69.4% (59.9%–77.8%)	88.5% (77.8%–95.3%)

Specificity, sensitivity, positive and negative predictive value of the non-invasive oscillometric blood pressure (NIBP) device in detecting a systolic and a mean invasive blood pressure lower than 80 mmHg and 60 mmHg at different cutoff limits. Data are expressed as percentage (95% confidential interval)

## Discussion

According to the criteria set at priori with mean bias lower than 10 mmHg and precision of 15 mmHg, only non-invasive MAP and DAP showed an acceptable agreement with the IBP technique. If the more restrictive criteria of AAMI standard were assessed, non-invasive MAP met the requirements of the analysis that included only animals with invasive MAP lower than 60 mmHg. The oscillometric device evaluated in this study showed a sensitivity of almost 92% in detecting values of mean IBP below 60 mmHg if the cutoff for the mean NIBP was set at 65 mmHg but specificity was 61%. Non-invasive SAP below 80 or 90 mmHg have a limited ability to identify systolic IBP lower than 80 mmHg. The trending ability of the NIBP device is poor considering the four-quadrant and the polar plot analysis and the concordance rate was below 90% for all the measured pressures.

The mean bias was decreased and the limit of agreement was smaller if data points for mean IBP below 60 mmHg were analysed rather than consider the overall values. The evaluation of this subgroup was performed to detect the accuracy of the NIBP monitor in a situation of low arterial blood pressure commonly referred as clinical relevant hypotension requiring a treatment. The bias for MAP in our study is smaller than SAP similarly to the results achieved by Barter and Epstein whom compare an oscillometric device with the carotid artery IBP [2]. Oscillometric method measure MAP as the pressure recorded at the point of maximal oscillation while SAP is usually estimated [9]. The bias for systolic blood pressure was lower if only invasive SAP values below 80 mmHg were considered and this may reflect an inadequacy of the device algorithm at high SAP values. Bias between NIBP and IBP measurements for DAP are almost identical as observed in other oscillometric device by Barter & Epstein [2].

Bland-Altman analysis, although widely used to evaluate the agreement between a monitoring device with a reference technique, does not provide any information on the ability of a new technology in detecting changes consistent with those measured by the “gold standard” technique. This characteristic is known as trending ability of a device. Recently an analytical method was introduced to assess the trending ability of new devices for cardiac output measurement and the same analysis was applied to arterial blood pressure monitoring [13–15]. The trending ability in our study resulted poor with a concordance rate below 90% for SAP, DAP and MAP. However no published studies define a limit for good trending ability for blood pressure monitors either for the four-quadrant or the polar plot analysis. The limits reported in our study to define acceptable concordance rate and trending ability were set on previous literature in human medicine that in turn uses limits to assess cardiac output monitors [13, 15]. Moreover the refinement of the exclusion zone by Receiver Operating Characteristic (ROC) curve analysis was recommended for cardiac output monitoring although literature is lacking in a suitable exclusion zone for trending ability of blood pressure monitor. The exclusion zone used in our study has been reported previously although an optimal value for NIBP monitor is not yet outlined [13, 15]**.** The trending ability in this study may also have been affected by a low agreement between measurement methods especially at high blood pressure. Minimal changes in IBP might be associated with opposite changes of high magnitude in NIBP. Those changes, included in the analysis, may have caused a dispersion of data in the four-quadrant and polar plot analysis affecting the concordance rate and the trending ability. Moreover hypotensive episodes in some rabbits responded poorly to a decrease in end-tidal isoflurane and as no other treatments were performed, the assessment of trending ability in our study might be difficult to quantify.

Rabbits are particularly sensitive to the vasodilatatory effects of volatile anaesthetics despite an ongoing surgical procedure or the application of a supramaximal electrical stimulation [4, 16]. Harvey et al. arbitrarily defined hypotension in anaesthetized rabbits as invasive SAP or MAP lower than 60 mmHg and/or 80 mmHg respectively [4], and based on these criterion most of the rabbits in the study were classified as hypotensive. Because mean bias between NIBP and IBP for MAP and SAP were almost 5 and 10 mmHg, it was decided to evaluate the performance of the device describing hypotension with a cutoff of 65 and 90 mmHg respectively. Using the cutoff of 65 mmHg for MAP the device showed a superior sensitivity in detecting mean IBP lower than 60 mmHg. The new analysis indeed showed an increase in sensitivity for MAP of almost 92% while the sensitivity to detect a systolic IBP lower than 80 mmHg remains lower regardless the cutoff considered, although it increases from 46 to 74%.

Our study presents some limitations: first the agreement between IBP and NIBP was evaluated only in the front limb, while hind limb may have resulted in a different outcome. Ypsilantis et al. demonstrated a good correlation between the oscillometric pressures measured on the front limb with that recorded on the abdominal aorta rather the one recorded on the hind limb [7]. For neutering procedure the positioning of the cuff on the hind limb was considered less suitable because it proves difficult to reach in case of dislodgment or detachment.

All the rabbits enrolled in this study were owned patients undergoing elective neutering. While the use of such cases may provide a good evaluation of the oscillometric device in a clinical setting, on the other hand this represents a limitation because these may have introduced confounding factors due to a different haemodynamic status based on the surgical stimulations.

Another limitation is that during the surgical procedure, the intensity of the cardiovascular stimulation may have varied rapidly on the base of the manoeuvre performed. The dynamic response test was performed at the beginning of each anaesthesia but it was not repeated during the surgical procedure. The fundamental frequency, represented by the pulse rate (170–200 beats/min), was almost 6 times the natural frequency measured. This contributed to reduce the underdamping of the fluid-filled pressure transducer system due to the low damping coefficient recorded in most of rabbits [10]. Underdamping of a system used to measure IBP tends to overestimate SAP while the opposite is observed in a system that demonstrates overdamping. It cannot be excluded that according to the increase in the haemodynamic status a change in the damping coefficient may have influenced the agreement particularly at high blood pressure values.

## Conclusion

Agreement is poor between the systolic arterial blood pressure measured with the non-invasive device and that measured invasively at the level of ear artery. Invasive MAP is overestimated at high pressure values although bias decreases if only measurements lower than 60 mmHg obtained invasively were considered and match the requirement of good agreement based on ACVIM and AACI guidelines. Trending ability of the new device is poor although for MAP concordance rate is superior than SAP and DAP. The sensitivity of the device is higher in detecting invasive MAP lower than 60 mmHg if a cutoff value of 65 mmHg is considered and may be used to identify hypotension in anaesthetized rabbits although it has a poor ability to identify false positive.

## Abbreviations

DAP: Diastolic arterial blood pressure
IBP: Invasive arterial blood pressure
IV: Intravenous
MAP: Mean arterial blood pressure
NIBP: Non-invasive arterial blood pressure
SAP: Systolic arterial blood pressure
